# Validation of the illustrated questionnaire on food consumption for Brazilian schoolchildren (QUACEB) for 6- to 10-year-old children

**DOI:** 10.3389/fpubh.2023.1051499

**Published:** 2023-09-22

**Authors:** Giovanna Angela Leonel Oliveira, Daniela Oliveira Llorente Barrio, Giovanna Soutinho Araújo, Marina Pimentel Saldanha, Raquel Machado Schincaglia, Muriel Bauermann Gubert, Natacha Toral

**Affiliations:** ^1^Graduate Program in Human Nutrition, Faculty of Health Science, Center for Epidemiological Studies in Health and Nutrition (NESNUT), University of Brasilia, Brasilia, Brazil; ^2^Department of Nutrition, Faculty of Health Science, Center for Epidemiological Studies in Health and Nutrition (NESNUT), University of Brasilia, Brasilia, Brazil; ^3^School of Public Health, University of Nevada, Las Vegas, NV, United States

**Keywords:** validation study, feeding pattern, diet, food intake, surveys and questionnaires, child, chronic diseases

## Abstract

**Introduction:**

Evaluating the food consumption of school-aged children is crucial to monitor their dietary habits, promote targeted interventions, and contribute public policies that aimed healthy eating. In this context, our objective was to develop and validate the Illustrated Questionnaire on Food Consumption for Brazilian Schoolchildren (QUACEB) of 6 to 10 years old, which is a self-reported illustrated recall.

**Methods:**

Validity was obtained in four stages as follows: selection of foods, validation of items, validation of illustrations, and pretest. Foods were selected by considering the data from the main surveys that have been conducted with the Brazilian population and schoolchildren in recent years, the degree of food processing, and the main foods from each of the country's five macroregions. The content of the items was validated by comparing the children's and their parent's responses. For this, the questionnaire was published in an online format, and 6- to 10-year-old elementary schoolchildren were recruited using the snowball technique. The first part of the questionnaire was answered by the parent after the child's lunch, and the second was completed by the child the following day. Thirty-two parent and child dyads participated. Sensitivity, specificity, area under the curve (AUC), and kappa (k) tests were performed.

**Results:**

Of the 30 foods presented on the questionnaire, 15 were reported as consumed. High sensitivity (mean of 88.5%), high specificity (average of 92.0%), substantial agreement (*k* = 0.78), low disagreement (6.2%), and AUC of 0.90 were found. The illustrations were validated in a focus group with fourth-grade children from a school chosen for convenience. The food illustrations were designed for children, who were asked to name the food. Eighteen children participated and verified that the images were representative of the foods. In the pretest, three schools were chosen for convenience that announced the link to the online questionnaire in WhatsApp groups of parents with students from first to fifth grade. Fifteen children answered the questionnaire and 86.7% (*n* = 13) judged it excellent or good.

**Conclusion:**

Thus, the food consumption questionnaire is valid for elementary schoolchildren of 6 to 10 years old and can be applied in research to assess the dietary patterns of children in Brazil.

## 1. Introduction

Childhood obesity has reached epidemic levels and is a modern public health problem (1, 2). In Brazil, data from the Food and Nutrition Surveillance System (SISVAN in Portuguese) indicate an unfavorable trend toward obesity in Brazilian children between 5 and 10 years old, with a prevalence of 10.45% and 16.96% in a temporal variation between 2008 and 2021, respectively (3). Although the etiology of childhood obesity is complex, poor diet is an important independent risk factor for the development of non-communicable diseases (NCDs) and obesity (4).

The data are related to changes in the quality of the Brazilian diet in recent years, marked by an increase in the consumption of ultra-processed foods (5). In general, ultra-processed foods are highly palatable, are low in fiber, contain excess sugar and/or sodium, and have high levels of total and saturated fats, which add greater energy value to the diet and could increase the risk of chronic diseases (6–8).

The unfavorable dietary nutrient profile of ultra-processed foods impacts the quality of the diet negatively and has direct consequences on health, and their consumption should be continuously evaluated and monitored at this stage of life (9). The most common methods to monitor the food consumption of Brazilian children are the Food Record, the 24-h Dietary Recall (R24 h), and the Food Frequency Questionnaire (10). Structured food consumption questionnaires, such as the R24 h, are a good option for studies that assess student health, as they are simple, practical, and low-cost methods (11).

Most of the tools developed to assess food consumption are not validated and are not intended for school-aged children, or the food list is outdated (12–14). Most of the recent questionnaires validated for school-aged children are from other countries, including Poland (15), Japan (16), Turkey (16), Malaysia (17), Lebanon (18), England (19), Spain (20), Chile (21), and Europe (22). Specifically in Brazil, instruments validated for school-aged children are rare. Studies with children from São Paulo (23, 24), Salvador (25), and Western Amazon (26) stand out. However, all of these questionnaires are semi-quantitative, and none are illustrated. In addition, most respondents are parents (15, 16, 18, 19, 21, 23, 24, 26, 27), but a few have a questionnaire applied directly to children under parental or teacher guidance (17, 18, 20, 22). Burrows et al. (28) showed that the Food Frequency Questionnaire reported by children of 8 to 12 years of age was the closest to the gold standard measure (doubly labeled water method) when compared with the report of children's food consumption by their parents.

However, especially for children aged 6 to 10 years, few instruments are available to collect information on food consumption, especially when the objective is for the child to be the informant. This is explained by the fact that the assessment of food consumption is challenging, considering that children in this age group are not able to provide reliable information on usual intake and serving size, in addition to requiring memory, attention span, motivation, and cognition (29).

The instruments proposed to fulfill this objective are the Previous Day Food Questionnaire (PDFQ) and the Food Consumption and Physical Activity Questionnaire for schoolchildren (Web-CAAFE). The PDFQ is a questionnaire designed for schoolchildren that uses an illustrated recall to qualitatively analyze food consumption on the previous day (30). However, the instrument does not include regional Brazilian foods. The presence of these foods is important to enhance the culture, habits, and food traditions. Web-CAAFE is a software for the qualitative measurement of food consumption through the recall of the previous day. The instrument includes more food options than the PDFQ, including regional ones. However, access is restricted and is through a system with login and password (31).

The importance of research that evaluates the food consumption of schools for carrying out epidemiological studies is undeniable. However, this assessment must be carried out with adequate, updated, and validated instruments, which consider the cognitive limitations of each age. Thus, the objective of this study is to validate an accessible qualitative questionnaire on food consumption for Brazilian children of 6 to 10 years of age.

## 2. Methods

We developed and validated an illustrated questionnaire to investigate the food consumption of elementary schoolchildren between 6 and 10 years of age. This is a quantitative and qualitative study and was carried out in four stages as follows: (1) selection of foods to develop the questionnaire; (2) validity test of the chosen foods by comparing the children's self-report and their parents' observation; (3) two focus groups with children to validate the illustrations; and (4) pretest.

The project was approved by the Ethics Committee on Research with Humans of the Faculty of Health Sciences of the University of Brasilia (Protocol CAAE 25866919.4.0000.0030). The parents or guardians agreed with the free and informed consent form and the children with the free and informed consent form.

The proposed questionnaire was given the acronym QUACEB, corresponding to the initials of the name in Portuguese: “*Questionário de Consumo Alimentar para Crianças Escolares Brasileiras*” (Illustrated Questionnaire on Food Consumption for Brazilian Schoolchildren).

### 2.1. Stage 1: QUACEB development

The questionnaire was created according to the following criteria: (a) The most consumed foods were included according to data from the Family Budget Survey (POF in Portuguese) 2017–2018 (32) and the 2015 National School-based Health Survey (PeNSE in Portuguese) (33); (b) To choose the foods, the food groups and the degree of food processing were considered, according to the Dietary Guidelines for the Brazilian Population (7); (c) Later, representative foods from all Brazilian macroregions were inserted (34, 35); and (d) The name of the food was added in uppercase letters as a caption for the figures. The figures were designed by a graphic designer specializing in products for children. Initially, 30 foods or food groups were included, among those most consumed according to the national surveys (**Table 2**).

### 2.2. Stage 2: tests to validate the foods in the QUACEB

Evidence of the validity of the foods illustrated was obtained through comparison tests between the parent's account (father, mother, or guardian) who observed the food consumed by the child after lunch and the child's self-report on the following day of the food they had eaten for lunch. The parents' report was considered a gold standard. We chose only one meal to make the instrument faster, simpler, and accessible. Lunch was selected because it is the most consumed meal for Brazilians (32).

The sample was selected for convenience and consisted of dyads of Brazilian parents and children, between 6 and 10 years of age, who had access to the Internet and enrolled in elementary schools in Brazil. The sample size was calculated based on kappa for 2 raters' estimation with an expected kappa (k) equal to 0.75, expected precision of 0.3, the proportion of the outcome (p) of 0.5 (considering the same probability of having or not having the binary outcome), and the confidence level of 99%. The sample size was calculated at 33, and we added 10% to cover drop-outs, totaling 37 dyads of parents and children. We used an online calculator available at https://wnarifin.github.io/ssc/sskappa.html (36). Study participants were recruited using the snowball methodology (37). The snowball is a sampling technique, and the participants recommend the survey to another individual in his network (37). For this, a research poster containing the QRCode for accessing the questionnaire link was prepared. Moreover, the poster was publicized on the researcher's and the university's social networks. In addition, disclosure was carried out in groups of WhatsApp parents from some schools known to the researchers.

On the first day, parents completed their questionnaire immediately after the child's lunch. The questionnaire for the guardians included four screens as follows: (1) the free and informed consent form; (2) identification of data about the child (date of birth, sex, and initial name); (3) characterization of the parent (gender, marital status, age, education, family income in minimum wages, and state residing), education network of the child's school and the child's school year; and (4) information on child's food consumption. The included questions are as follows: whether the child had lunch that day; a list of 30 foods for the parent to mark which of these the child consumed at lunch; and an open-ended question to write down any foods that were consumed but were not on the list.

At the end of the questionnaire, the parent was instructed that the child should answer the questionnaire the next day, in the morning, without any interference.

The child's questionnaire contained three screens as follows: (1) data to identify the child (date of birth, gender, and initials); (2) term of consent of the minor; and (3) information on food consumption. This screen asked what the child had eaten for lunch the day before and contained 30 illustrations of food with captions for the child to select which ones were consumed at lunch the day before, for better understanding and adaptation to the age group.

The researchers used the collection of child identification data in both questionnaires to aggregate the responses obtained on the collection for 2 days and identify the respective parent–child dyads. The questionnaire was accessible for 31 days from September to October 2020.

The responses to the questionnaires automatically generated a database in Microsoft Office Excel format, which was exported to use for the analysis. The tests were performed using the MedCalc software, adopting a significance level of 5%. Data are presented in absolute (n) and relative (%) frequencies.

For the external validity of the questionnaire, the values of sensitivity (the ability to detect the consumption actually presented, i.e., true positives divided by the sum of true positives and negatives), specificity (the ability to indicate no consumption when there were actually none presented, i.e., true negatives divided by the sum of true negatives and false positives), the area under the curve (AUC), and their respective 95% confidence intervals (95% CI) were calculated using the parents' report as the gold standard. The closer the AUC value is to 1, the better the instrument performed (38). Kappa statistics (k) with its 95% CI were also calculated to assess the agreement between the responses of the parent and the child, considering *k* = 0 as an absence of agreement; *k* between 0.41 and 0.60 for moderate agreement; *k* between 0.61 and 0.80 for substantial agreement; *k* between 0.81 and 0.99 for almost perfect agreement; and *k* = 1 for perfect agreement (39).

### 2.3. Step 3: focus groups to substantiate the validity of QUACEB illustrations

To validate whether the illustrations were representative of the food, two focus groups were conducted in October 2021 with fourth-grade children (9 years old) from a public school in the Federal District, Brazil, chosen for convenience and the fourth-grade class was chosen by the school principal. Two sessions were held in the classroom, and each session lasted for ~50 min. In the second session, reached information saturation. The participants included nine children in each session and were conducted by three researchers (one moderator and two observers). The sessions were audio-recorded with the children's permission.

The group sessions were organized according to the following steps: presentation of the researchers and the research; clarification of the dynamics of participatory discussion and request for consent for participation and audio recording; presentation of illustrations and individual active listening; and ending by thanking them. The group dynamics to assess students' understanding of the figures occurred through the projection on a classroom wall of 43 illustrations without captions (33 food illustrations after modifications based on the results of the earlier validity test and 10 more regional food groups in Brazil). The group moderator provided the following guidance: “Let's play a guessing game. I'm going to show you some pictures and I would like you to tell me the names of what you see.” Then, each figure of food was presented separately. Next, they were asked: “Do you recognize this food? And what is the name of this food?”. After the question, it was advised that the child lifts his hand to tell the name of the food being projected. To ensure that everyone participates, we randomly chose a few children to say what they observed. The participants were free to discuss among themselves and actively listen.

### 2.4. Stage 4: QUACEB pretest

The Illustrated Questionnaire on Food Consumption for Brazilian Schoolchildren was built on the Google Forms platform, with items, writing, and illustrations modified according to the results of the previous stages. In addition, information on the age and gender of the child was included, as well as the number of meals eaten on a typical day and possible foods consumed the previous day which were not included in the questionnaire. Three schools were chosen for convenience, one public, located in the Federal District, Brazil, and two-thirds of the sample coming from private schools located in a small municipality in the State of Goiás, Brazil. The schools were contacted *via* telephone and agreed to publicize the research link in the WhatsApp groups of parents in the elementary school (from the first to the fifth school year). The questionnaire link along with a link to publicize it was provided to the schools. The link was available for 20 days in November 2021. The pretest was applied to test the application format, i.e., whether children could fill out the online questionnaire alone or under their guardian's supervision just to help read the questions. For this, an orientation was written for parents to deliver their cell phones or computers for children to fill out the questionnaire alone, and whether necessary adults can ask them the questions without interfering with the child's answers. At the end of the questionnaire, the children were asked what they thought of the questionnaire, with response options on a five-point Likert scale (ranging from 1 “great” to 5 “very bad”), and children were also asked to fill in an open question for suggestions to improve the questionnaire.

## 3. Results

**Figure 1:**
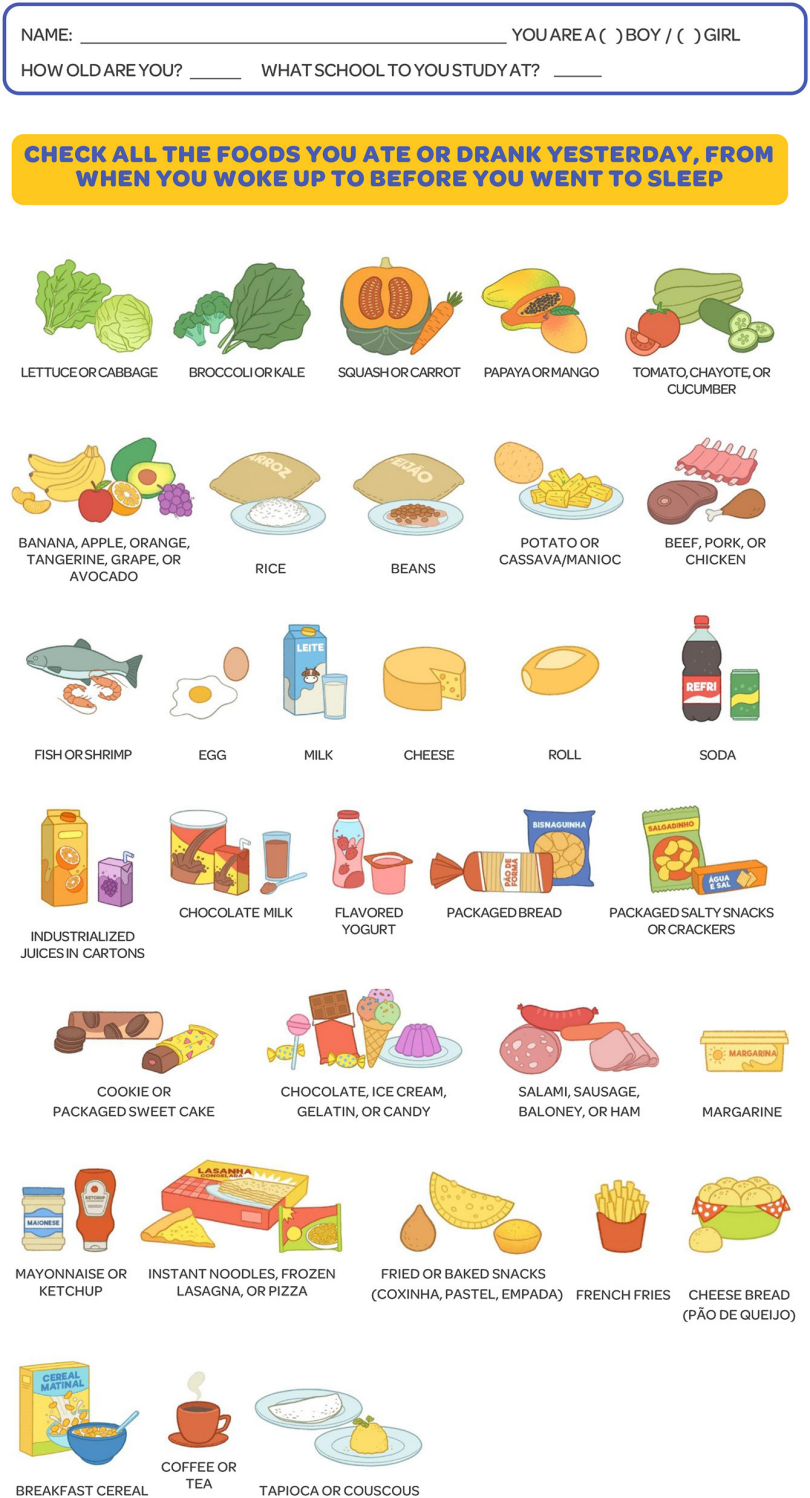
Illustrated Questionnaire on Food Consumption for Brazilian Schoolchildren (QUACEB), 2021.

**Figure 2:**
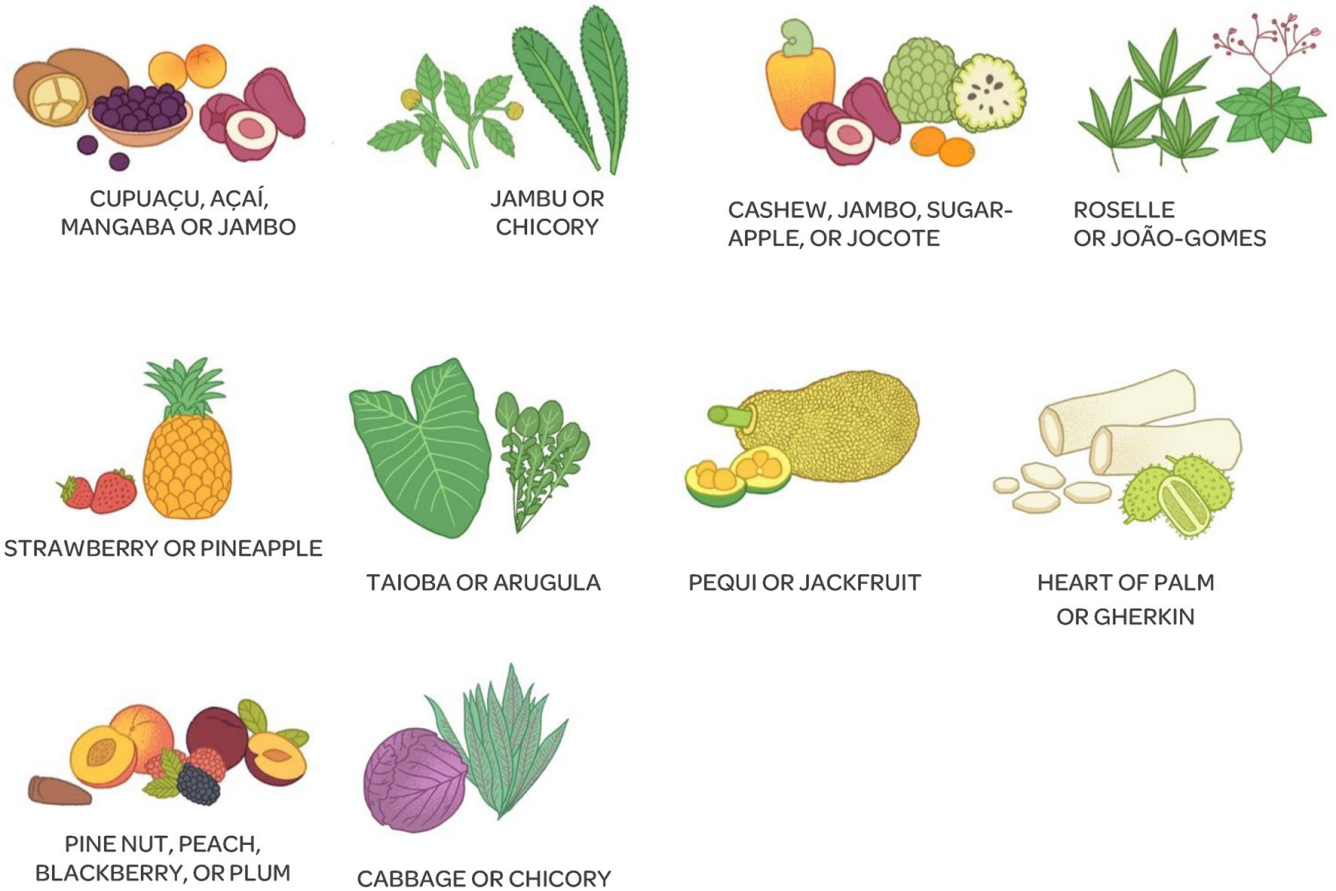
Additional module with figures of regional foods that can be inserted in the Illustrated Questionnaire on Food Consumption for Brazilian Schoolchildren (QUACEB).

As a result of the four stages, the instrument was developed for an illustrated self-reported recall, intended for 6- to 10-year-old Brazilian children. It contained a list of 33 groups of national food figures (Figure 1), with the option of adding 10 illustrations of regional fruits and vegetables (Figure 2).

In the online study to validate the content of QUACEB items (the second stage of the study), 32 parent–child dyads participated, most of them from the Federal District (59.38%). Most of the parents were women (93.75%), between 35 and 54 years old (71.87%), had a graduate degree or more (56.25%), were married/in a stable relationship (68.75%), and with a monthly family income above 10 minimum wages (equivalent to R$ 10,450.00 or U$ 2,061) (53.12%). Half of the children were girls (50.00%) and most studied in private schools (78.13%) (Table 1), with a mean age of 8 ± 0.85 years.

**Table 1:** Research stage to validate the content of the Illustrated Questionnarie on Food Consumption for Brazilian Schoolchildren (QUACEB), 2021.

**Study variables**	***n***	**%**
**Place of residence**		
Federal District	19	59.38
Minas Gerais	2	6.25
Rio de Janeiro	1	3.13
São Paulo	1	3.13
Tocantins	9	28.13
**Parent's gender**		
Female	30	93.75
Male	2	6.25
**Age of the parent**		
19 to 34 years old	8	25.00
35 to 44 years old	15	46.87
45 to 64 years old	9	28.13
**Education of the parent**		
Incomplete higher education or less	5	15.63
Complete higher education	9	28.12
Postgraduate degree	18	56.25
**Marital status of the parent**		
Married/stable union	22	68.75
Single/divorced	10	31.25
**Family income (in minimum wages** ^*^ **)**		
up to 3	5	15.63
3 to 6	4	12.50
6 to 10	6	18.75
More than 10	17	53.12
**Child's gender**		
Female	16	50.00
Male	16	50.00
**Child's age (Years)**		
6	2	6.25
7	9	28.13
8	14	43.75
9	7	21.88
**Child's school type**		
Private	25	78.13
Public	7	21.87
**Child's grade in school (year)**		
1^st^	5	15.63
2^nd^	6	18.75
3^rd^	18	56.25
4^th^	3	9.38

^*^Minimum Wage at the time of the survey was R$1,045.00, equivalent to U$206.00.

Source: compiled by authors.

Of the 30 food groups initially listed in the instrument, stage 1 of the QUACEB development elucidated that only 15 had a minimum consumption frequency that would allow statistical tests for validation. Comparisons of the responses between children and their guardians indicated frequent consumption (more than 37.5%) of rice, beans, beef/pork/chicken, juice, and lettuce/tomato. Of the food groups that had low consumption (<3.13%), 10 were not mentioned by parents or children (nuggets/hamburger/pizza/instant noodles, coffee, milk, cookie/packaged sweet cake, breakfast cereal, packaged bread, rolls, couscous/tapioca, cheese bread/coxinha/pig in blanket, and snack chips); three groups were reported only by children (cheese, chocolate milk boxed or powdered/industrialized yogurt, and salami/sausage/baloney/ham); one group was reported only by parents (mango/papaya); and one group was reported by both parents and children (soup) (Table 2).

**Table 2:** Analysis of disagreement, sensitivity, specificity, area under the curve, and kappa between the reports of children and their parents participating in the validation survey of the Illustrated Questionnarie on Food Consumption for Brazilian Schoolchildren, 2021.

**Food**	**Report (%)**		**Disagreement (%)**	**Sensitivity CI_95%_**	**Specificity CI_95%_**	**AUC CI_95%_**	**Kappa CI_95%_**
	**Child**	**Parent**					
Rice	81.25	81.25	6.16	96.15 (80.4–99.99)	83.33 (35.9–99.6)	0.90 (0.74–0.98)^*^	0.79 (0.52–1.00)^*^
Beans	75.00	71.88	3.13	95.83 (78.9–99.99)	100.00 (63.1–100.00)	0.98 (0.85–1.00)^*^	0.92 (0.77–1.00)^*^
Toasted manioc	9.38	9.38	6.16	66.67 (9.40–99.20)	96.55 (82.20–99.90)	0.82 (0.64–0.93)^¥^	0.63 (0.16–1.00)^*^
Lettuce/tomato	37.50	37.50	12.50	83.33 (51.60–97.90)	90.00 (68.30–98.80)	0.87 (0.70–0.96)^*^	0.73 (0.49–0.98)^*^
Broccoli/chayote/ kale	12.50	12.50	0	100.00 (39.8–100.00)	100.00 (87.70–100.00)	1.00 (0.89–1.00)^*^	1.00 (1.00–1.00)^*^
Squash/carrot/ pequi	18.75	25.00	12.50	83.33 (35.90–99.60)	88.46 (69.80–97.60)	0.86 (0.69–0.96)^¥^	0.63 (0.31–0.96)^*^
Beef/pork/ chicken	75.00	84.38	9.38	100.00 (85.80–100.00)	62.50 (24.50–91.50)	0.81 (0.64–0.93)^*^	0.71 (0.42–1.00)^*^
Egg	9.38	9.38	0	100.00 (29.20–100.00)	100.00 (88.10–100.00)	1.00 (0.89–1.00)^*^	1.00 (1.00–1.00)^*^
Fish/shrimp	6.25	6.25	0	100.00 (29.20–100.00)	100.00 (88.10–100.00)	1.00 (0.89–1.00)^*^	1.00 (1.00–1.00)^*^
Soup	3.13	3.13	0	BC	BC	BC	1.00 (1.00–1.00)^*^
Nuggets/ hamburger/ pizza/ instant noodles	0	0	0	BC	BC	BC	BC
Soda	6.25	3.13	3.13	50.00 (1.30–98.70)	100.00 (88.40–100.00)	0.75 (0.57–0.88)^¥^	0.65 (0.02–1.00)^*^
Juice	53.13	53.13	25.00	76.47 (50.10–93.20)	73.33 (44.90;92.20)	0.75 (0.56–0.88)^**^	0.50 (0.20–0.80)^**^
Coffee	0	0	0	BC	BC	BC	BC
Sweets	9.38	12.50	3.13	100.00 (29.20–100.00)	96.55 (82.20–99.90)	0.98 (0.86–1.00)^*^	0.84 (0.53–1.00)^*^
Milk	0	0	0	BC	BC	BC	BC
Cheese	3.13	0	3.13	BC	BC	BC	0.00 (0.00–0.00)^€^
Chocolate milk boxed or powdered/ industrialized yogurt	3.13	0	3.13	BC	BC	BC	0.00 (0.00–0.00)^€^
Cookie/packaged sweet cake	0	0	0	BC	BC	BC	BC
Breakfast cereal	0	0	0	BC	BC	BC	BC
Packaged bread	0	0	0	BC	BC	BC	BC
Roll	0	0	0	BC	BC	BC	BC
Apple/grape/ banana/orange	6.25	9.38	3.13	100.00 (15.80;100.00)	96.67 (82.80;99.90)	0.98 (0.86;1.00)^*^	0.78 (0.38–1.00)^*^
Pasta	9.38	12.50	3.13	100.00 (29.20–100.00)	96.55 (82.20–99.90)	0.98 (0.86–1.00)^*^	0.84 (0.53–1.00)^*^
Couscous/Tapioca	0	0	0	BC	BC	BC	BC
Cheese bread/coxinha/pig in blanket	0	0	0	BC	BC	BC	BC
Salami/sausage/ baloney/ham	3.13	0	3.13	BC	BC	BC	0.00 (0.00–0.00)^€^
mango/papaya	0	3.13	3.13	BC	BC	BC	0.00 (0.00–0.00)^€^
Cassava/manioc/ potato	12.50	12.50	6.26	75.00 (19.40–99.40)	96.43 (81.70–99.90)	0.86 (0.69–0.95)^**^	0.71 (0.34;1.00)^*^
Snack chips	0	0	0	BC	BC	BC	BC
All			6.24	88.45	92.02	0.90	0.78

AUC, area under the curve; CI_95%_- 95% confidence interval; LC, low consumption reported and observed (statistical tests could not be performed).

^*^p < 0.001; ^**^p < 0.01; and ^¥^ p > 0.05; ^€^*p*-value not obtained due to insufficient sample.

Source: completed by authors.

There was a low disagreement between the answers of the parents and children, with an average of 6.2% and a variation from 0 (broccoli/chayote/kale, egg, fish/shrimp, and soup) to a maximum of 25.0% (juice) (Table 2).

Sensitivity values, i.e., the probability that the children reported what they actually ate as presented by their parents, indicate an average of 88.5% for all food groups. The lowest sensitivity value (50.0%) was found in the soda group and maximum values (100.0%) occurred in the broccoli/chayote/kale, beef/pork/chicken, egg, fish/shrimp, sweets, apple/grape/banana/orange, and pasta groups (Table 2).

The specificity values (average of 92.02%) demonstrated that the questionnaire was able to detect foods that were not consumed when, in fact, there was no consumption. The beef/pork/chicken group had the lowest value for specificity (62.50%), while the beans, broccoli/chayote/kale, egg, fish/shrimp, and soda groups had the highest values (100%) (Table 2).

The indices of the area under the curve (AUC) were employed to verify the global accuracy of the questionnaire, as this parameter considers the simultaneous analysis of the specificity and sensitivity measures for each food item. As shown in Table 2, the egg and fish/shrimp food items had maximum values of sensitivity and specificity and, thus, the highest values for AUC (1.00). On the other hand, the soda and juice groups had the lowest value (0.75). The mean of the 15 groups analyzed was 0.90, indicating the good performance of the instrument for these food items.

The kappa test between the child's and the parent's reports was significant for all items that presented satisfactory consumption for validation (more than 6.25% consumption), with an average of *k* = 0.78 (38). Of the 15 food items analyzed, 7 groups (beans, broccoli/chayote/kale, egg, fish/shrimp, soup, sweets, and pasta) had an “almost perfect or perfect” agreement (*k* ≥ 0.81) and only juice obtained a kappa value with moderate classification (*k* = 0.41–0.60) (Table 2).

Based on the results found for the validity of the items, the following changes were made to the questionnaire. Ten illustrations of regional foods were added—five fruits and five vegetables from each Brazilian macroregion (northern region—cupuaçu/açaí/mangaba/jambo and jambu/chicory; northeast—cashew/jambo/sugar-apple/jocote and roselle/João-Gomes; southeast —strawberry/pineapple and taioba/arugula; central-west—pequi/jackfruit and heart of palm/herkin; and south—pine nut/peach/blackberry/plum and cabbage/chicory). This consequently caused the pequi to be removed from the squash and carrot group; reformulation of groups of raw vegetables, including tomato and chayote, in a specific grouping with cucumber; inclusion of groups of dark green vegetables, including broccoli and kale, and of light green vegetables, containing lettuce and cabbage. Toasted manioc, soup, nuggets, hamburgers, açaí preparation, popsicle, pasta, salami, and pigs in blanks were removed to make fewer items on the list, along with tangerine and avocado in the fruit grouping, potato chips, mayonnaise and ketchup, margarine, frozen lasagna, tea, fried pastry, and baked pastry. With these changes, QUACEB now contains 43 illustrations of food groups, 33 of which are national food groups and 10 are regional food groups.

In the focus group for the illustration validation stage, the 43 updated illustrations were presented to the children. The figures that were difficult to recognize were raw cassava/manioc, jocote, açaí, and mangaba, due to the disproportionate size of the food in the drawing and the group of pine nuts. Thus, the students gave suggestions to improve the images, which were accepted, and the drawings were redone. Therefore, in the final version of QUACEB, a bowl of boiled cassava/manioc was used; jocote, açaí, and mangaba were resized; and only one pine nut was captured. Some images of regional foods including the heart of palm, gherkin, taioba, jambu, chicory, roselle, and João-Gomes were not recognized because children were unaware of the food itself. During the focus groups, the children also presented suggestions for the captions. From this, the following changes were made to the caption: the description of the term “pão de sal” was included in the image of rolls; including the two terms “*biscoito*” and “*bolacha*” (both of which regional words for cookie) in the corresponding image; and the nomenclature of industrialized yogurt was changed to flavored yogurt and from boxed chocolate to chocolate milk. With the focus groups, we concluded that the students satisfactorily understood most of the images, and the need to change some figures and legends was raised, providing final improvements to the questionnaire.

In the pretest, 15 children who participated had a mean age of 9 years ± 1.13, mostly boys (66.7%), 8 of whom studied in the public school and 7 in the two private schools. Most reported an average consumption of 4 ± 0.80 meals per day, and all reported eating breakfast and lunch. Of the 33 national food groups listed, 29 had a frequency of consumption reported on the previous day. Among these, the most consumed were beef, pork, or chicken (86.67%); rice (80.00%); beans (66.67%); and milk (66.67%). The least consumed foods (6.67%) were broccoli or kale; egg; packaged salty snacks or crackers; instant noodles, frozen lasagna or pizza; fried or baked snacks (coxinha, pastel, and empada); French fries; and cheese bread. Regarding regional foods, only one child (6.67%) reported consumption of the following groups: cupuaçu, açai, mangaba, or jambo; and pine nuts, peaches, blackberries, or plums (Table 3). Four children recorded the consumption of other foods that were not on the list, namely, water, cotton candy, cheese cracker, and macaroni. Regarding the evaluation of the questionnaire, 86.7% of the participating children judged the questionnaire as excellent or good and did not register possible suggestions.

**Table 3:** Frequency of food consumption reported by children participating in the QUACEB pre-test of Brazil, 2021.

**Food groups**	**Frequency**	
	***n***	**%**
Lettuce or cabbage	3	20.00
Broccoli or kale	1	6.67
Squash or carrot	4	26.67
Papaya or mango	0	0.00
Tomato, chayote, or cucumber	4	26.67
Banana, apple, orange, tangerine, grape or avocado	7	46.67
Rice	12	80.00
Beans	10	66.67
Potato or cassava/manioc	2	13.33
Beef, pork, or chicken	13	86.67
Fish or shrimp	0	0.00
Egg	1	6.67
Milk	10	66.67
Cheese	4	26.67
Roll	8	53.33
Soda	6	40.00
Industrialized juices in cartons	3	20.00
Chocolate milk	8	53.33
Flavored yogurt	0	0.00
Packaged bread	2	13.33
Packaged salty snacks or crackers	1	6.67
Cookie or packaged sweet cake	4	26.67
Chocolate, ice cream, gelatin, or candy	7	46.67
Salami, sausage, baloney, or ham	6	40.00
Margarine	5	33.33
Mayonnaise or ketchup	4	26.67
Instant noodles, frozen lasagna, or pizza	1	6.67
Fried or baked snacks (coxinha, pastel, and empada)	1	6.67
French fries	1	6.67
Cheese bread	1	6.67
Breakfast cereal	3	20.00
Coffee or tea	5	33.33
Tapioca or couscous	0	0.00
Cupuaçu, açaí, mangaba, or jambo fruits	1	6.67
Jambu or chicory	0	0.00
Cashew, jambo, sugar-apple, or jocote	0	0.00
Roselle or João-Gomes	0	0.00
Strawberry or pineapple	0	0.00
Taioba or arugula	0	0.00
Pequi or jackfruit	0	0.00
Heart of palm or gherkin	0	0.00
Pine nut, peach, blackberry, or plum	1	6.67
Cabbage or chicory	0	0.00

## 4. Discussion

The present study demonstrated that the illustrated Questionnaire on Food Consumption for Brazilian Schoolchildren (QUACEB) was valid for schoolchildren. Overall, the instrument achieved good performance, according to the sensitivity, specificity, kappa, and AUC indices, in addition to having a low discordance value. Furthermore, the graphic representations proved to be understandable and attractive to the children.

Currently, validated and illustrated questionnaires with children from southern Brazil in which the child is the respondent include the Previous Day Food Questionnaire (PDFQ) (31, 40, 41); the Typical Day of Physical Activity and Food Intake (DAFA) questionnaire (42) and its electronic version—the WEBDAFA (43); and the Food Intake and Physical Activity of School Children (Web-CAAFE) (31, 44). PDFQ and DAFA contain a list of foods that are repeated in the six daily meals (breakfast, morning snack, lunch, afternoon snack, dinner, and bedtime snack). The difference is that the QUADA assesses the consumption of the previous day and the DAFA of a regular day (30, 42). WEBDAFA has the same structure as the printed instrument but is hosted on a website; however, the interface is currently not available (43). Web-CAAFE has been enhanced from the PDFQ and DAFA experience, is a recall of the day before hosting, and is hosted on a website. However, it is necessary to register in the system to issue a password, and currently, the system only monitors schools in the municipal education network of Florianopolis (31).

Another aspect considered necessary for a food consumption assessment questionnaire for schoolchildren is that it allows the analysis of consumption according to the degree of food processing. The NOVA classification, adopted in the Dietary Guidelines for the Brazilian Population (7), has already been widely described in the literature as important for public health, considering that the consumption of ultra-processed foods has been associated with several chronic diseases at different stages of life (6, 45, 46). The quantitative questionnaire considered the extent and purpose of food processing to assess the usual diet of schoolchildren of 9 to 10 years of age constructed by Amorim et al. (47). The results showed that the children in the study were able to respond without the support of their parents. However, the instrument has not yet been validated, is restricted to children in a very narrow age group, and is not illustrated (47).

Among the ultra-processed foods evaluated, the consumption of industrialized juices, sweets, and soda was observed at lunch. The other foods in this category that were initially included in the list of 30 food groups in the questionnaire had no consumption reported by the participants. A study that evaluated the intake of ultra-processed foods in 105 schoolchildren of 7 to 10 years of age from a public district located in Teresina, Piaui, highlights the participation of these three groups in the list of the ultra-processed foods most consumed by the public evaluated (45). This again reinforces the need for instruments such as the questionnaire developed and validated here, which can detect food consumption according to the degree of processing (30, 48).

Although there are no population studies in Brazil with children aged 5 to 9 years, other studies find similar patterns of food consumption among schoolchildren. For example, a study carried out in a Brazilian municipality in 2007, with children aged 7 to 10 years, found that the most consumed foods at lunch and dinner were rice, beef or poultry, beans, soft drinks, and pasta (49). In another study conducted in Brazil in 2017, with children aged 7 to 13 years, the foods that had the highest average daily frequency of consumption were rice, bread, beef/chicken, and beans (50). Furthermore, the foods most commonly consumed by the Brazilian population are rice, beans, beef or poultry, and bread (32). In this study, the most consumed foods were beef, pork or chicken, rice, beans, and milk.

It is important to emphasize that comparisons between the results for the validation of this questionnaire with other validated instruments are limited due to methodological differences, especially in the reference method used and the age group covered. Even so, the kappa values were similar to the validation results of the last version of PDFQ (30), obtaining the same value of the agreement test for the fruit group (*k* = 0.78). Other foods, such as the meat and pasta group, were also similar in both studies for this variable, with *k* = 0.69 and *k* = 0.81 being found in PDFQ, respectively, for the items mentioned and *k* = 0.71 and *k* = 0.84 obtained in this validation.

The kappa value was lower for the juice group, and it had the greatest disagreement in responses between the reports of the children and their parents. The data found highlight a discrepancy, which could be the different interpretations of this drink for parents and children within the analyzed group. The caption of this group “juice” could be interpreted as encompassing various preparations, such as fresh juice, industrialized juice, and concentrated drink. The initial illustration only contained an image of a box of juice, which could be interpreted exclusively as this type of preparation. Thus, we understand that a more specific description of this item and the adequacy of the illustration were necessary to reduce different interpretations and produce better levels of agreement.

The developed questionnaire is inexpensive and easy to apply, which has been demonstrated in previous studies with similar questionnaires (30, 43). The computerized format saves application time, eliminates interviewer-related biases, and ensures automated storage of collected information. Few validated online instruments are available that assess food consumption, especially for school-aged children (29, 30, 43).

The access of Brazilian students to new information and communication technologies has increased in significant proportions (51), which makes online self-report instruments useful and promising to assess the eating habits of the public. Studies state that the application of an online questionnaire is a promising alternative that helps to keep children's attention on the research (52, 53).

Another advantage of the developed and validated questionnaire is that, unlike traditional paper questionnaires, the online format enables data collection from different Brazilian regions. This will allow the inclusion of multiple cultural spheres and facilitate the generalization of results to the general population in future studies with a larger sample size. The possibility of including fruits and vegetables typical of all Brazilian regions, such as pequi, jackfruit, avocado, the heart of palm, and gherkin from the central-west; cashew, jambo, jambu, sugar-apple, jocote, roselle, and João-Gomes from the northeast; cupuaçu, açaí, mangaba, jambo, jambu, and chicory from the north; strawberry, pineapple, avocado, taioba, and arugula from the southeast; and pine nut, peach, blackberry, plum, cabbage and chicory from the south, is also noteworthy to allow the regionalization of the questionnaire with the inclusion of regional foods (33, 34).

This work differs from other validation studies of children's questionnaires, as it proposes to evaluate children's food consumption when they are outside the school context, both in person and online, considering the classification of foods according to the degree of processing recommended by the Dietary Guidelines for the Brazilian Population (7), allows the inclusion of regional foods, and describes the food in the caption. Thus, the instrument can serve as support material for future epidemiological studies of health and nutrition and nutritional intervention programs for this age group.

When using QUACEB, food consumption can be analyzed according to different markers, for example by the NOVA score (46), the classification proposed by the Dietary Guidelines for the Brazilian Population (41), the food diversity score (54), markers of protective foods and risk of excess body fat (55–58), nutritional profile represented by nutrient sources in food groups (19), food-based classification of eating episodes (FBCE) (42, 59), identifying dietary patterns (60), or describing the consumption of regional foods.

The weaknesses of the developed and validated questionnaire include the lack of information about the portion size or the possibility of estimating the child's energy consumption. However, it allows a qualitative assessment of children's consumption, in a brief and straightforward way, in which the researcher can provide reliable data on food consumption, seeking to avoid biasing the child's memory. Other limitations of this work include the lack of presentation of internal validity, only external due to the small sample size and the presence of foods with infrequent consumption, but the most frequent foods were validated and this would possibly happen for other foods; the absence of sensitivity, specificity, AUC, and kappa analyzes by gender and age, due to the insufficient sample; and the lack of exploration of factors associated with disagreements due to the low prevalence and sample size. Furthermore, validation occurred only by testing a meal and a 1-day period. Thus, future studies should be carried out with a larger sample, all meals of the day (to assess consumption of items not reported at lunch), and also test–retest style analysis, whereby participants fill it in for a number of multiple days to see how accurate it is over 1 day. Despite acknowledging that children within the study's age group lack purchasing power and parents are conscious of their children's dietary consumption, we recommend that future validation studies incorporate direct observation throughout the entire day to assess the child's food consumption.

In conclusion, the illustrated online food consumption questionnaire demonstrated adequate concordance, sensitivity, specificity, and area under the curve values to assess the Illustrated Questionnaire on Food Consumption for Brazilian Schoolchildren of 6- to 10-year-old when compared with the parents' report. The QUACEB is a valid, simple, brief, practical, easy-to-apply questionnaire available on the Internet in any Brazilian region, which can be adopted for epidemiological research to assess the diet of that population. This tool is specifically designed to be appropriate to Brazil because it represents the foods most consumed by Brazilian schoolchildren.

## Data availability statement

The raw data supporting the conclusions of this article will be made available by the authors, without undue reservation.

## Ethics statement

The studies involving humans were approved by Ethics Committee on Research with Humans of the Faculty of Health Sciences of the University of Brasilia (Protocol CAAE 25866919.4.0000.0030). The studies were conducted in accordance with the local legislation and institutional requirements. Written informed consent for participation in this study was provided by the participants' legal guardians/next of kin.

## Author contributions

NT and MG worked on the project design. GO, MS, DB, and GA participated in data collection and analysis. RS performed the statistical analyses. All authors helped with the data interpretation and writing of the article, reviewed and approved the final version, and verified that they were responsible for all aspects of the study in guaranteeing the accuracy and integrity of any part of the study.
