# Supplementary material for: Validation of the illustrated questionnaire on food consumption for Brazilian schoolchildren (QUACEB) for 6- to 10-year-old children
Source: Front Public Health. 2023 Sep 22;11:1051499. doi: 10.3389/fpubh.2023.1051499 (PMC10559973; doi:10.3389/fpubh.2023.1051499)
Supplement: Supplementary file 4 [file Data_Sheet_3.PDF]

NOME: ____ **VOCÊ É ( ) MENINO / ( ) MENINA**

QUANTOS ANOS VOCÊ TEM?______ **EM QUAL ANO DA ESCOLA VOCÊ ESTÁ ESTUDANDO?**______

MARQUE TODOS OS ALIMENTOS QUE VOCÊ COMEU OU BEBEU ONTEM, DESDE QUANDO ACORDOU ATÉ ANTES DE DORMIR

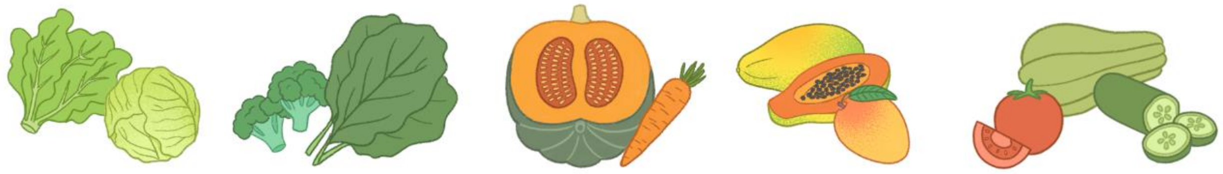

ALFACE OU REPOLHO | BRÓCOLIS OU COUVE | ABÓBORA OU CENOURA | MAMÃO OU MANGA | TOMATE, CHUCHU OU PEPINO

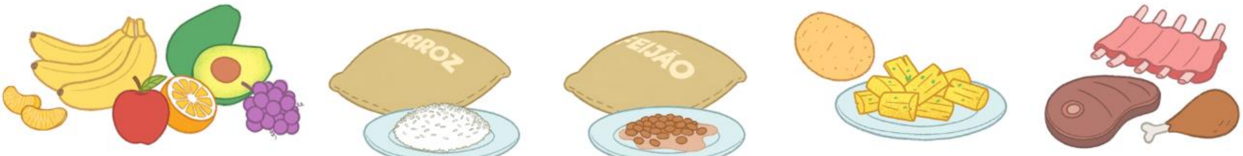

BANANA, MAÇÃ, LARANJA, TANGERINA, UVA OU ABACATE | ARROZ | FEIJÃO | BATATA OU MANDIOCA | CARNE DE BOI, PORCO OU FRANGO

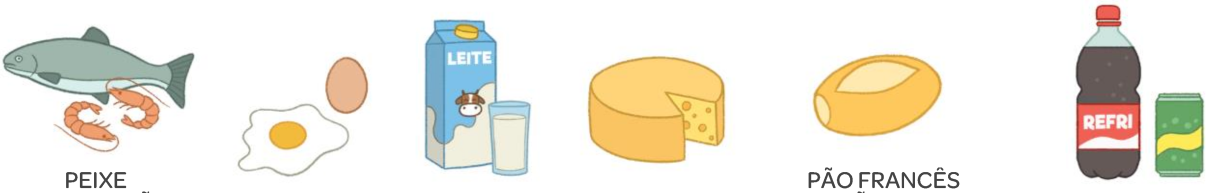

PEIXE OU CAMARÃO | OVO | LEITE | QUEIJO | PÃO FRANCÊS OU PÃO DE SAL | REFRIGERANTES

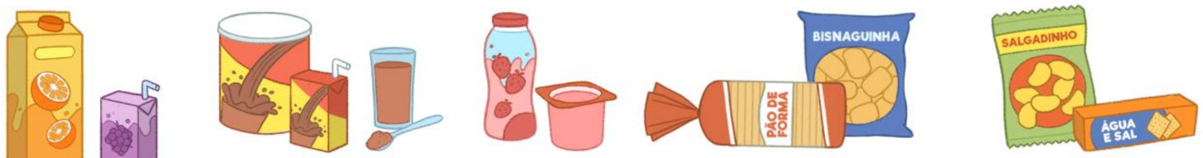

SUCO DE CAIXINHA | BEBIDA ACHOCOLATADA | IOGURTE COM SABOR | PÃO DE FORMA OU BISNAGUINHA | SALGADINHO DE PACOTE OU BISCOITO/BOLACHA SALGADO

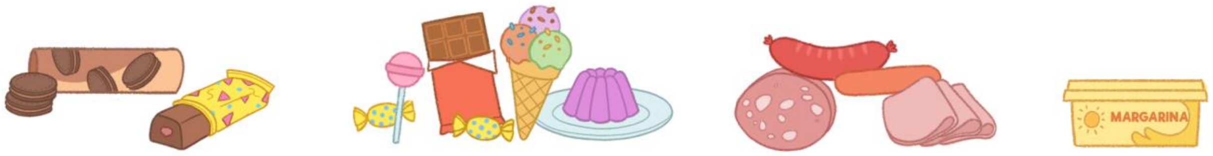

BISCOITO/BOLACHA RECHEADO OU BOLINHO DE PACOTE | CHOCOLATE, SORVETE, GELATINA, PIRULITO OU BALINHA | SALSICHA, MORTADELA, LINGUIÇA OU APRESUNTADO | MARGARINA

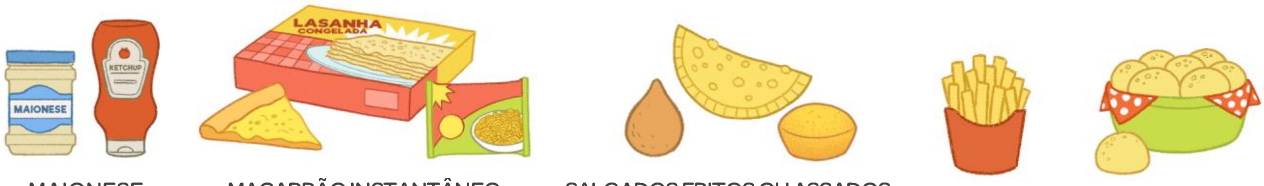

MAIONESE OU KETCHUP | MACARRÃO INSTANTÂNEO, LASANHA CONGELADA OU PIZZA | SALGADOS FRITOS OU ASSADOS (COXINHA, PASTEL, EMPADA) | BATATA FRITA | PÃO DE QUEIJO

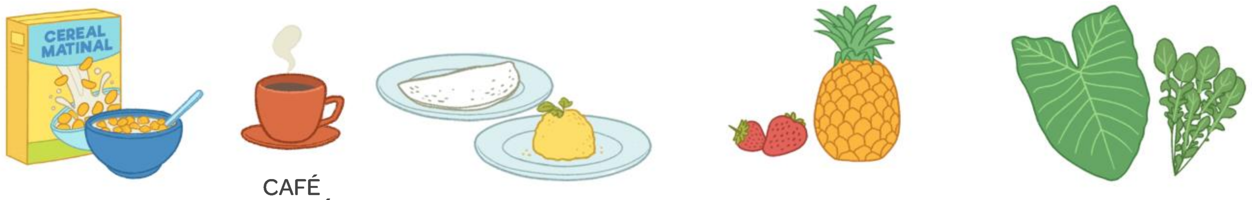

CEREAL MATINAL | CAFÉ OU CHÁ | TAPIOCA OU CUSCUZ | MORANGO OU ABACAXI | TAIOBA OU RÚCULA
