# Supplementary material for: Validation of the illustrated questionnaire on food consumption for Brazilian schoolchildren (QUACEB) for 6- to 10-year-old children
Source: Front Public Health. 2023 Sep 22;11:1051499. doi: 10.3389/fpubh.2023.1051499 (PMC10559973; doi:10.3389/fpubh.2023.1051499)
Supplement: Supplementary file 5 [file Data_Sheet_4.PDF]

NOME: ____ VOCÊ É ( ) MENINO / ( ) MENINA

QUANTOS ANOS VOCÊ TEM?_____ EM QUAL ANO DA ESCOLA VOCÊ ESTÁ ESTUDANDO?_____

MARQUE TODOS OS ALIMENTOS QUE VOCÊ COMEU OU BEBEU ONTEM, DESDE QUANDO ACORDOU ATÉ ANTES DE DORMIR

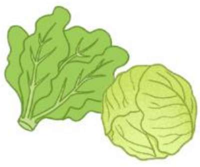
ALFACE OU REPOLHO

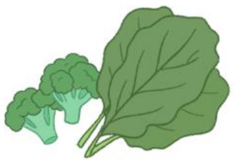
BRÓCOLIS OU COUVE

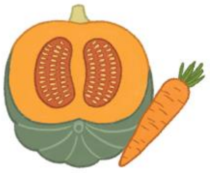
ABÓBORA OU CENOURA

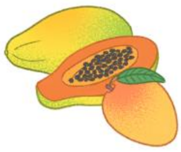
MAMÃO OU MANGA

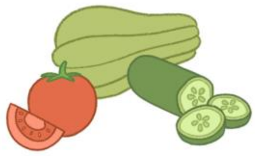
TOMATE, CHUCHU OU PEPINO

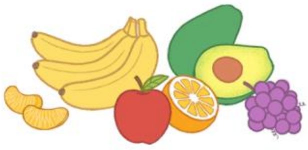
BANANA, MAÇÃ, LARANJA, TANGERINA, UVA OU ABACATE

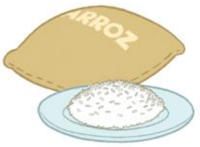

ARROZ

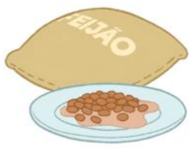

FEIJÃO

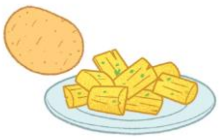
BATATA OU MANDIOCA

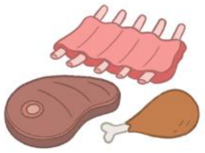
CARNE DE BOI, PORCO OU FRANGO

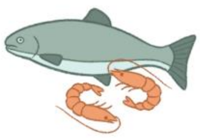
PEIXE OU CAMARÃO

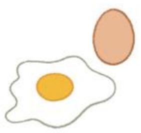
OVO

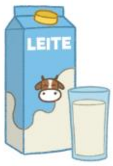

LEITE

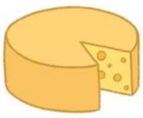
QUEIJO

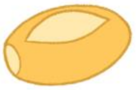
PÃO FRANCÊS OU PÃO DE SAL

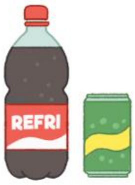

REFRIGERANTES

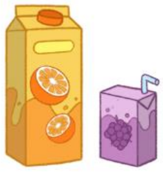
SUCO DE CAIXINHA

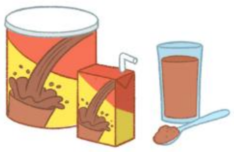
BEBIDA ACHOCOLATADA

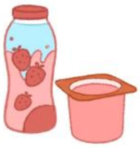
IOGURTE COM SABOR

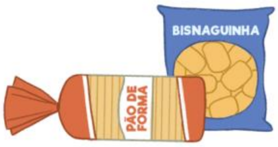

PÃO DE FORMA OU BISNAGUINHA

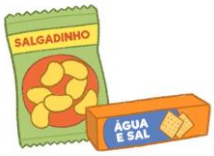

SALGADINHO DE PACOTE OU BISCOITO/BOLACHA SALGADO

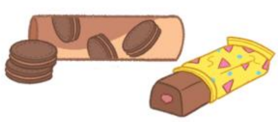
BISCOITO/BOLACHA RECHEADO OU BOLINHO DE PACOTE

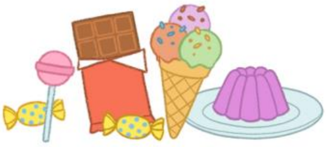
CHOCOLATE, SORVETE, GELATINA, PIRULITO OU BALINHA

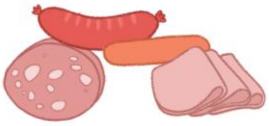
SALSICHA, MORTADELA, LINGUIÇA OU APRESUNTADO

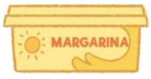

MARGARINA

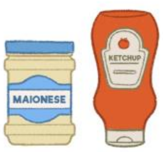

MAIONESE OU KETCHUP

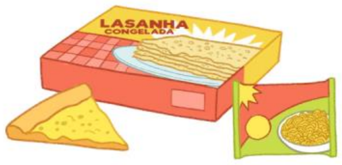

MACARRÃO INSTANTÂNEO, LASANHA CONGELADA OU PIZZA

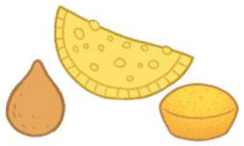
SALGADOS FRITOS OU ASSADOS (COXINHA, PASTEL, EMPADA)

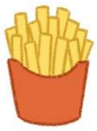
BATATA FRITA

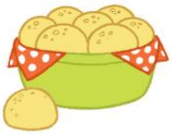
PÃO DE QUEIJO

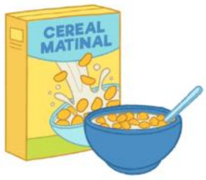

CEREAL MATINAL

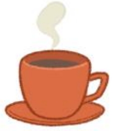
CAFÉ OU CHÁ

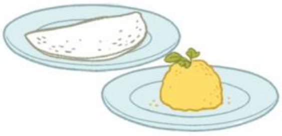
TAPIOCA OU CUSCUZ

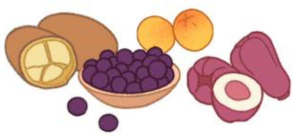
CUPUAÇU, AÇAÍ, MANGABA OU JAMBO

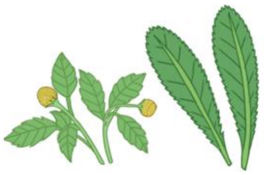
JAMBU OU CHICÓRIA-DO-PARÁ
